# Dopamine and the Creative Mind: Individual Differences in Creativity Are Predicted by Interactions between Dopamine Genes DAT and COMT

**DOI:** 10.1371/journal.pone.0146768

**Published:** 2016-01-19

**Authors:** Darya L. Zabelina, Lorenza Colzato, Mark Beeman, Bernhard Hommel

**Affiliations:** 1 Medical Social Sciences, Northwestern University, Chicago, IL, United States of America; 2 Leiden University, Institute for Psychological Research & Leiden Institute for Brain and Cognition, Leiden, the Netherlands; 3 Psychology Department, Northwestern University, Evanston, IL, United States of America; Catholic University of Sacro Cuore, ITALY

## Abstract

The dopaminergic (DA) system may be involved in creativity, however results of past studies are mixed. We attempted to clarify this putative relation by considering the mediofrontal and the nigrostriatal DA pathways, uniquely and in combination, and their contribution to two different measures of creativity–an abbreviated version of the Torrance Test of Creative Thinking, assessing divergent thinking, and a real-world creative achievement index. We found that creativity can be predicted from interactions between genetic polymorphisms related to frontal (COMT) and striatal (DAT) DA pathways. Importantly, the Torrance test and the real-world creative achievement index related to different genetic patterns, suggesting that these two measures tap into different aspects of creativity, and depend on distinct, but interacting, DA sub-systems. Specifically, we report that successful performance on the Torrance test is linked with dopaminergic polymorphisms associated with good cognitive flexibility and medium top-down control, or with weak cognitive flexibility and strong top-down control. The latter is particularly true for the originality factor of divergent thinking. High real-world creative achievement, on the other hand, as assessed by the Creative Achievement Questionnaire, is linked with dopaminergic polymorphisms associated with weak cognitive flexibility and weak top-down control. Taken altogether, our findings support the idea that human creativity relies on dopamine, and on the interaction between frontal and striatal dopaminergic pathways in particular. This interaction may help clarify some apparent inconsistencies in the prior literature, especially if the genes and/or creativity measures were analyzed separately.

## Introduction

Creative thought underlies not only many innovations in science, technology, and the arts, but also novel solutions to common problems people encounter in their daily lives. As a fundamental aspect of human cognition, creative thinking has recently emerged as an important topic in cognitive neuroscience, as witnessed by the sharp increase in publications on the subject in traditionally cognitive and neuroscience journals [1]

Previous studies have pointed to the involvement of the dopaminergic (DA) system in creativity [2–4]. Takeuchi et al. [4], for example, found individual differences in creativity, as measured by divergent thinking tests (in which people generate ideas in response to verbal or figural prompts), to be positively correlated with grey matter in DA system regions, including the dorsolateral prefrontal cortex, bilateral basal ganglia, substantia nigra, and the ventral tegmental area. Several genetic studies have shown a relationship between divergent thinking and dopamine neurotransmission [5–7]). Reuter et al. [7], for instance, found divergent thinking to be significantly associated with polymorphisms of the dopamine D2 receptor gene (DRD2). Participants with higher divergent thinking scores, particularly flexibility scores, were reported to carry the DRD4-7R allele [5]. Ideational fluency (but not originality) of divergent thinking was found to be linked with DAT, COMT, DRD4, and TPH1 [8]. Additionally, both verbal fluency and verbal originality were related to DAT x COMT, as well as to COMT x DRD4 interactions (although these interactions were not followed by post-hoc tests, therefore the nature of the interactions remains unclear) [9]. Moreover, various aspects of divergent thinking, such as verbal and figural fluency and flexibility were reported to relate to several polymorphisms of the COMT and DRD2 genes, as well as to their three- and four-way interactions [10].

Several studies have also found cortical dopamine to be involved in cognitive flexibility [11–13]–one of the main components of creativity in general and of creative thinking in particular [14]. Finally, higher levels of openness to experience and schizotypy, personality traits associated with creativity [15–17], have been linked with the activity in the dopaminergic system [18–20].

These findings suggest that DA is related to creativity, but this relation is not yet well understood. At least two characteristics of dopaminergic functioning might be responsible for the lack of clear understanding of the link between DA and creativity. First, there is not just one DA system, but three major pathways, with at least two of them putatively involved in regulating creative thinking and behavior [21]: the mediofrontal pathway originating in the ventral tegmental area, and the nigrostriatal pathway originating in the substantia nigra [12, 22]. The interaction between the mediofrontal and the nigrostriatal pathways has been assumed to be involved in cognitive control, and in the regulation of the balance between cognitive stability (e.g., maintaining a particular goal, focusing, concentrating) and cognitive flexibility (e.g., switching between different options, opening up for new information) [21], which is key for creative behavior [23]. The second characteristic that makes it difficult to understand how DA affects creative behavior is that the function relating dopaminergic production and performance in creativity tasks is not linear. For instance, Akbari Chermahini and Hommel [24] investigated the relationship between individual spontaneous eye blink rate, a marker of striatal DA, and performance in a divergent-thinking task. Performance was significantly better with medium than with low or high blink rates, suggesting that performance follows an inverted U-shaped function. Understanding the impact of DA on creativity thus requires the assessment of the mediofrontal and the nigrostriatal DA pathways in ways that allow one to look into different levels of dopaminergic functioning. In the present study, we tried to do this by considering two genes that tap into the frontal and the striatal dopaminergic pathways, and by considering polymorphisms of these genes that are assumed to be associated with different levels of dopaminergic functioning.

**Fig 1:**
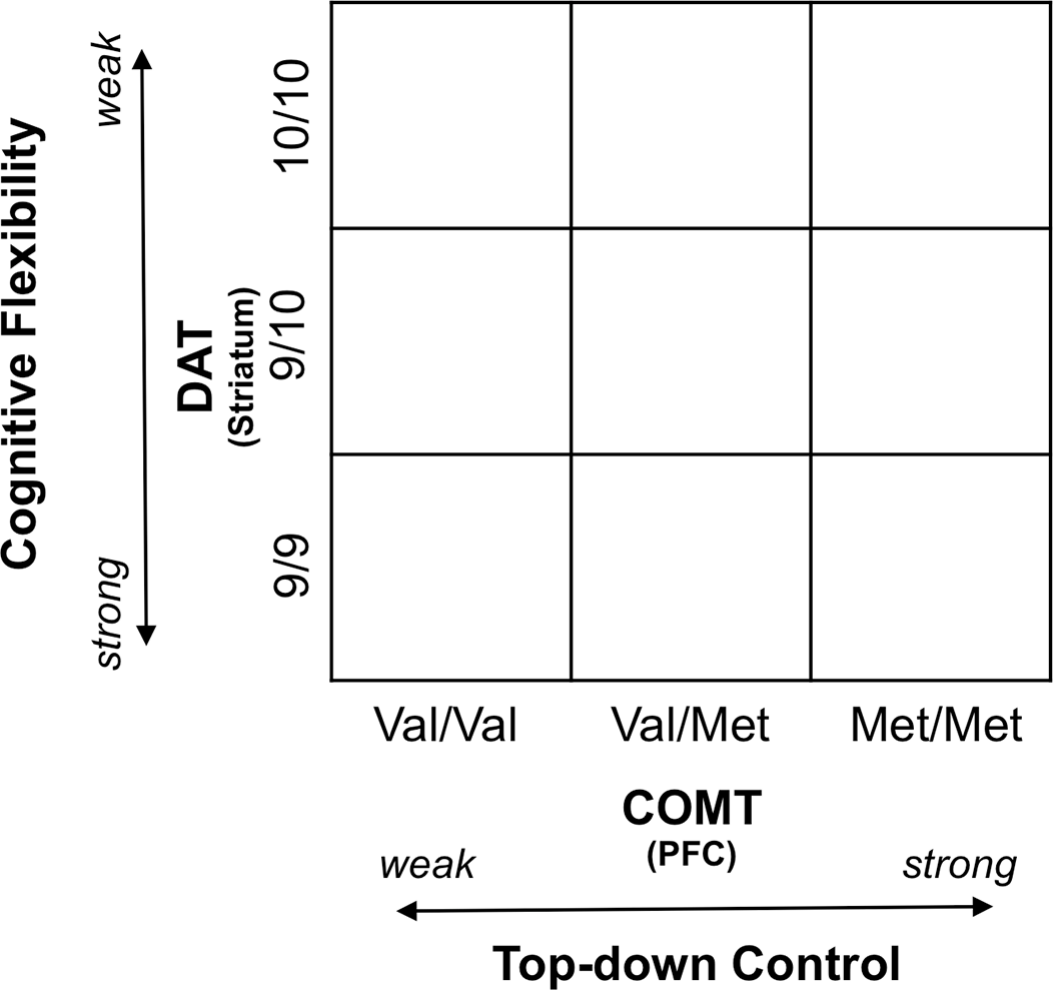
Putative associations between COMT (tied to DA availability in the prefrontal DA pathways) and top-down cognitive control; and DAT (tied to DA availability in striatal pathways) and cognitive flexibility.

The first DA gene we considered codes for the enzyme COMT, the most important mechanism for dopamine degradation in the prefrontal cortex. It contains a functional polymorphism (Val^158^Met) influencing enzyme activity [25] that affects cognition and behavior, and that plays a role in several neuropsychiatric disorders [26, 27]. Although the COMT Val^158^Met polymorphism has not been consistently found to increase risk of neuropsychiatric disorders [27], several studies found evidence for an association between the low-activity *Met* allele and improved performance on cognitive tasks involving the prefrontal cortex (PFC) in the healthy population [28–30]. COMT has been reported to affect dopamine synthesis, and to modulate dopaminergic interactions between the PFC and the midbrain [30]. Of particular importance, human studies have shown that the ValVal allele is associated with worse performance in executive function and working memory [29, 31], with inefficient frontal activation [28, 29], and the greater levels of noise in prefrontal circuits [32]. In the following, we will assume that the genetic setup of Met carriers is more supportive of cognitive top-down control than the genetic setup of Val carriers, with ValMet carriers falling in between (see Fig 1).

The second gene we considered was the DA transporter (DAT) gene, which is responsible for DA reuptake in the striatum [33]. Genetic variation of the DAT gene is associated with individual variation in the availability of DA transporters and striatal DA levels. This was confirmed by Positron Emission Tomography and by Single-Photon Emission Computed Tomography reporting the 10-repeat allele to be associated with lower availability of striatal dopamine transporters (thus to higher available dopamine levels) than the 9-repeat allele [34, 35]. While a smaller sampled in vivo study proposed the opposite [36]. Bertolino at al. [37] suggested that the 9-repeat allele of the DAT (much like the COMT Val allele) is linked with a selective decrease in tonic DA subcortically, by producing an activation of phasic DA transmission, while the 10-repeat allele (like the COMT Met allele) would increase tonic DA and decrease phasic DA subcortically. Because of contradictory findings [34–36] regarding whether 10-repeat allele or 9-repeat allele is indeed associated with lower availability of striatal DA transporters (linked to higher dopamine levels), predictions based on the amount of available DA are difficult. Functionally, the picture is clearer. The 9-repeat allele has been found to be positively related to reward-seeking behavior [38], greater sensitivity to change, and, in some cases, more distractibility [13], even though other studies have found less distractibility [39, 40], while the 10-repeat allele has been reported to be related to the opposite phenomena: low learning abilities and ADHD [41]. Accordingly, we interpreted DAT to tap into the dimension of cognitive flexibility. In particular, we will assume that the genetic setup of 9-repeat carriers is more supportive of cognitive flexibility than the genetic setup of 10-repeat carriers, with 9/10 carriers falling in between (see Fig 1).

Creativity is also a complex, and there exist different types of creativity, as well as different ways of measuring it. One of the most commonly used measures of creativity is a test of divergent thinking termed the Abbreviated Torrance Test for Adults (ATTA; [42]). Divergent thinking tests ask participants to generate multiple original ideas within a limited amount of time in laboratory settings [42, 43]. Responses are scored in terms of fluency (number of ideas), originality (statistical novelty of responses), and flexibility (number of categories). An alternate way to measure creativity is to simply catalogue people’s creative achievements in the real world. Such creative achievement questionnaires are surveys on which people indicate their achievement in various creative domains, encompassing theater, creative writing, scientific inventions, and other domains [44]. Here we used these two measures to evaluate creativity, and to investigate whether they have differential relationship to DA neurotransmission.

While divergent thinking and real-world creative achievement are typically modestly correlated [45– 47], and there is a link between divergent thinking and everyday creative activities, which in turn predict actual creative achievements [48], our previous investigations suggest that there are fundamental differences in how real-world creative achievers and people who perform well on divergent thinking tests attend to environmental stimuli and process sensory information. We find that ATTA performance is linked with cognitive and attentional mechanisms that sub-serve executive functions [47], i.e., general-purpose control mechanisms supported by the prefrontal cortex [49, 50]. Moreover, people with high ATTA scores (controlling for academic achievement) also exhibit selective sensory gating, i.e., they are successful at inhibiting “irrelevant” or repetitive sensory information, as assessed by the P50 ERP [46]. Conversely, people with high real-world creative achievements appear to have broad or “leaky” attention [47], as well as leaky sensory gating, as assessed by the P50 ERP [46]. Indeed for creativity leaky attention may help people notice information that is outside their focus of attention, and integrate this information into their current information processing, leading to a creative thought. This mechanism is akin to reduced latent inhibition, or reduced ability to screen or inhibit from conscious awareness stimuli that were previously experienced as irrelevant [51]. In other words, reduced latent inhibition may enhance creativity by enlarging the range of unfiltered stimuli available in conscious awareness, thereby increasing the odds of synthesizing novel and useful combinations of stimuli [52]. Although creative achievement is an outcome rather than a process, our previous findings suggest that there may be fundamental underlying differences between people who perform well on divergent thinking tests and people who achieve in creative domains in the real world. Thus, different brain systems may contribute to a different degree to different processes involved in the two measures, and therefore may be differentially associated with dopamine related genes.

The main question underlying the present study was how to explain the individual differences that these creativity assessments indicate. Specifically, we investigated whether performance on the ATTA and the real-world creative achievement measure can be explained through interactions between genes tapping into frontal and striatal dopaminergic pathways. If so, good performance on the divergent thinking test and creative achievement questionnaire should be associated with particular combinations of COMT and DAT polymorphisms. To the degree that these combinations would differ, we would need to conclude that different dopaminergic systems are linked with divergent thinking and creative achievement.

In addition to individual differences in DA-related genetic predispositions and aspects of everyday creativity, we also considered academic tests scores (Scholastic Assessment Test (SAT; [53]) or American College Testing (ACT; [54]), which are heavily influenced by intelligence as assessed by the WAIS-III [55], as a proxy for intelligence, which would likely also be related to the measures of creativity [56, 57]. Our sample was rather small for a behavioral genetics study, thus we consider our conclusions preliminary.

## Methods

### Participants

One hundred healthy young adults ages 18–30 (mean age = 20.57, SD = 2.49, male/female = 33/67) took part in the present study. Participants were recruited via advertisements posted at Northwestern University, and received $20 for their participation. None of the participants abused alcohol or drugs, and none smoked. None of the participants had been hospitalized for psychiatric or neurologic reasons. Four participants had history of depression or mild anxiety (three in the past, but in remission at the time of the study and not taking medication; one current, treated with Zoloft). Given that there are ethnic differences in the allele frequencies of both the COMT and DAT genes we tested only Caucasian participants [58, 59]. All subjects were right-handed, as assessed by the Chapman Handedness Questionnaire [60]. All participants provided their written informed consent prior to participating. The study was approved by the Institutional Review Board of Northwestern University.

### Measures and Procedures

Participants were tested individually, with each session lasting up to two hours (as part of a larger study). Participants completed the divergent thinking test (ATTA), provided their SAT or ACT scores, as well as answers to a questionnaire surveying their creative achievements in the real world, and then provided their saliva samples.

#### Divergent Thinking

Participants completed the *Abbreviated Torrance Test for Adults* (ATTA; [42]), a shortened form of the Torrance Test of Creative Thinking [61]. The ATTA consists of three activities (3 minutes each), one involving verbal (written) responses (e.g., generating problems that may arise from being able to walk on air or fly without being in an airplane or a similar vehicle), and two involving figural responses (e.g., using incomplete figures to make pictures). Responses were scored for fluency (i.e., a count of the number of pertinent responses), and originality (i.e., the number of responses that are not typically produced, according to normative data); scores were summed across the three activities [42]. In addition to computing the fluency and originality scores, we computed the total ATTA score, which reflects a weighted score of fluency plus two times originality, to equally weight the two scores (since the average fluency score (14.46) was approximately double the average originality score (7.30); see [62] for suggestions on scoring creativity tests). Four participants did not complete the ATTA. The average score was 29.06 (*SD* = 8.04, range 15–50).

#### Real-world creative achievement

We assessed real-world creative behavior with the *Creative Achievement Questionnaire* (CAQ) [44], on which subjects identified prior achievements in ten creative domains (visual art, music, dance, architectural design, creative writing, humor, inventions, scientific discovery, theater and film, and culinary arts). In the Music domain, for example, questions range from “I have no training or recognized talent in this area” (score of 0) to “My compositions have been critiqued in a national publication” (score of 7). In the Scientific Discovery domain, items vary from “I have no training or recognized ability in this field” (score of 0) to “My work has been cited by other scientists in national publications” (score of 7). The CAQ has good test-retest reliability, internal consistency, shows predictive validity against artists’ ratings, and shows discriminant validity with the tests of IQ [44]. Separate domain scores were combined to form a single index of creative achievement.

In addition to calculating the overall creative achievement scores, we also computed participants’ creative achievement within artistic and scientific domains separately, following Carson and colleagues [44]. Our sample consisted of participants mostly with creative achievements in the artistic domains (*M* = 10.36, *SD* = 9.58, range 0–48) than participants with creative achievements in scientific domains (*M* = 3.64, *SD* = 5.25, range = 0–34), which is to be expected in a sample of undergraduate students mostly in their first or second year of college. The mean total creative achievement score was 14.00 (*SD* = 11.55, range 0–48).

#### Academic Achievement Scores

As a proxy for general intelligence, participants provided their SAT and/or ACT scores, which we converted into percentile scores based on the national statistics for all test-takers in 2012 (*M* = 98.00, *SD* = 2.23, range 87–100). In prior studies in our lab, self-reported scores were confirmed with actual scores through the admissions office, and the two correlated *r* = .97 [63]. Only 70 participants provided their SAT/ACT scores. The range of scores was quite narrow, so this measure should be interpreted cautiously.

### DNA Laboratory Analysis

Genomic DNA was extracted from saliva samples using the Oragine^TM^ DNA collection kit. The SNP Val^158^Met of the COMT gene (rs#4680) and the DAT gene (rs#28363170) was genotyped using PCR-RFLP techniques. All genotypes were scored by two independent readers by comparison to sequence-verified standards. For COMT Val^158^Met three genotype groups were established: Val/Val homozygotes, Val/Met heterozygotes and Met/Met homozygotes. For DAT gene three genotype groups were established: 9/9, 9/10, and 10/10 repeat carriers. Genotype groups 9/9 and 9/10 were combined into a single 9/- group due to low number of participants.

### Analytical Strategy

We first computed Pearson’s correlation coefficients to assess the relationship between ATTA fluency, ATTA originality, and ATTA total scores, CAQ arts, CAQ science, and CAQ total scores, and SAT/ACT scores. We then performed two separate 2 (DAT: 9/-, 10/10) x 3 (COMT: ValVal, ValMet, MetMet) analyses of variance (ANOVAs) on participants’ ATTA total scores, and CAQ total scores, first with, and then without academic achievement scores as a covariate, in order to examine the association between DA and divergent thinking and creative achievement. We followed these tests with the post hoc comparisons using the Tukey’s HSD test. Similar analyses were performed excluding participants with mood disorders. Additionally, we conducted 2 x 3 analyses of covariance (ANCOVAs) on participants’ ATTA fluency, ATTA originality, CAQ arts, and CAQ science domains scores. As the CAQ data are generally positively skewed (as they were in our case), we log-transformed total CAQ scores, and again performed a 2 x 3 ANCOVA on the log-transformed creative achievement scores, with academic achievement scores as a covariate. Finally, we computed a multivariate analysis of covarariance (MANCOVA) with the ATTA and CAQ as DVs, and COMT and DAT as IVs, with SAT/ACT scores as a covariate, to reduce for multiple testing.

## Results

Pearson’s correlations between variables of interest are reported in Table 1. As can be seen from the table, ATTA fluency and ATTA total scores were reliably associated with the SAT/ACT scores. ATTA originality showed a reliable association with the CAQ arts domains. There was no reliable association between ATTA total and CAQ total scores.

Levene’s test for homogeneity of variance proved to be non-significant: ATTA *p* = .55, and CAQ *p* = .48.

**Table 1:** Correlation matrix and descriptive statistics of ATTA fluency, ATTA originality, and ATTA total scores, CAQ arts, CAQ science, and CAQ total domain scores, and SAT/ACT scores.

	ATTA Fluency	ATTA Originality	ATTA Total	CAQ Arts	CAQ Science	CAQ Total	SAT/ACT
**ATTA Fluency**	——	.26*	.66**	.09	.00	.08	.29*
**ATTA Originality**		——	.90**	.25*	-.08	.17	.18
**ATTA Total**			——	.23*	-.06	.17	.27*
**CAQ Arts**				——	.14	.89**	.04
**CAQ Science**					——	.57**	.16
**CAQ Total**						——	.09
**Mean**	14.46	7.30	29.06	10.36	3.64	14.00	98.00
**SD**	3.71	3.12	8.04	9.58	5.25	11.55	2.23

*Note. ATTA = Abbreviated Torrance Test for Adults; CAQ = Creative Achievement Questionnaire. *p < .05

**p < .01.

**Fig 2:**
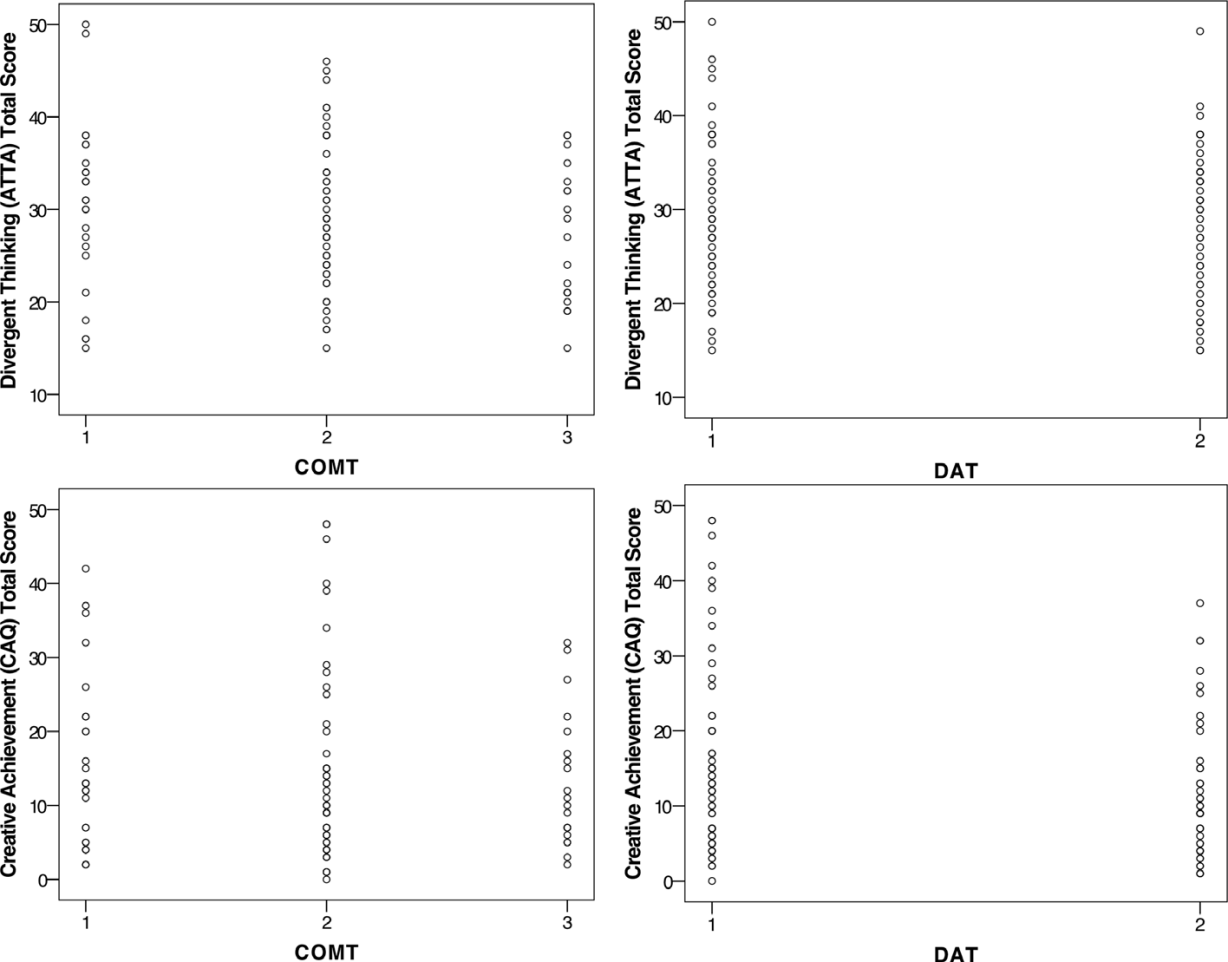
Scatterplots of ATTA and CAQ scores by COMT and DAT groups.

Prior to conducting the main analyses, we ensured that there were no outliers in the ATTA or CAQ scores by COMT and DAT groups (Fig 2).

### Results Involving ATTA Performance and Dopaminergic Polymorphisms

**Fig 3:**
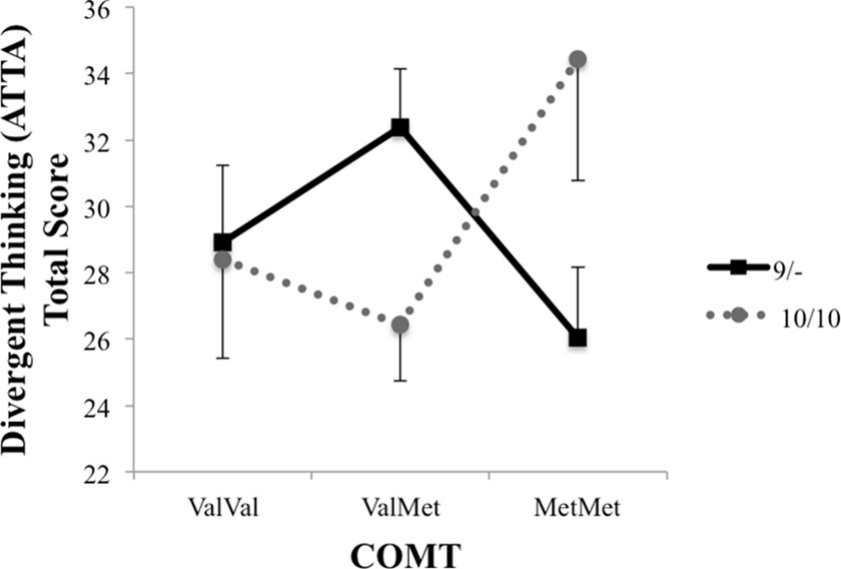
Estimated marginal means for ATTA total scores, controlling for academic achievement.

A 2 (DAT: 9/-, 10/10) x 3 (COMT: ValVal, ValMet, MetMet) ANCOVA was calculated on participants’ ATTA scores, with academic achievement scores as a covariate. There were no main effects for striatal (DAT) or frontal (COMT) dopamine, *p*s > .75, but there was a significant DAT x COMT interaction, *F*(2, 67) = 4.28, *p* = .02, partial Eta^2^ = .12. As can be seen in Fig 3, participants with the DAT 10/10 allele and COMT Met/Met allele, reflecting weak cognitive flexibility and strong top-down control, had the highest ATTA scores (*M* = 34.45, *SE* = 3.68). Post hoc comparisons using the Tukey’s HSD test indicated no reliable differences between the groups. A less stringent post hoc test using LSD Student’s *t*-test indicated that the mean ATTA score for the 9/-, MetMet group (*M* = 26.04, *SE* = 2.12) was reliably different from the 9/-, ValMet group (*M* = 32.37, *SE* = 1.78); and the ATTA score for the 10/10, ValMet group (*M* = 26.43, *SE* = 1.69) was reliably different from the 9/-, ValMet group (*M* = 32.37, *SE* = 1.78).

We also performed a 2 (DAT: 9/-, 10/10) x 3 (COMT: ValVal, ValMet, MetMet) ANOVA on participants’ ATTA scores *without* the academic achievement scores as a covariate. There were no main effects for striatal (DAT) or frontal (COMT) dopamine, *p*s > .48, and no significant DAT x COMT interaction, *p* > .18, indicating that the SAT/ACT factor introduces some variability that masks the interaction. Means and standard deviations for divergent thinking (not controlling for academic achievement) are presented in Table 2.

**Table 2:** ATTA total score means and standard deviations for the COMT and DAT groups.

COMT	DAT	Mean	SD	N
**ValVal**	9/-	30.64	9.12	14
	10/10	30.55	9.37	11
	Total	30.60	9.04	25
**ValMet**	9/-	30.92	7.67	26
	10/10	27.56	7.53	25
	Total	29.27	7.71	51
**MetMet**	9/-	25.14	6.76	14
	10/10	30.00	8.20	6
	Total	26.60	7.36	20
**Total**	9/-	29.35	8.10	54
	10/10	28.69	8.05	42
	Total	29.06	8.04	96

Similar analyses were performed excluding participants with the mood disorders (*N* = 4), with the DAT x COMT interaction becoming more robust, *F*(2, 63) = 5.12, *p* = .009, Eta^2 =^ .15. There were no main effects, *p*s > .75.

**Fig 4:**
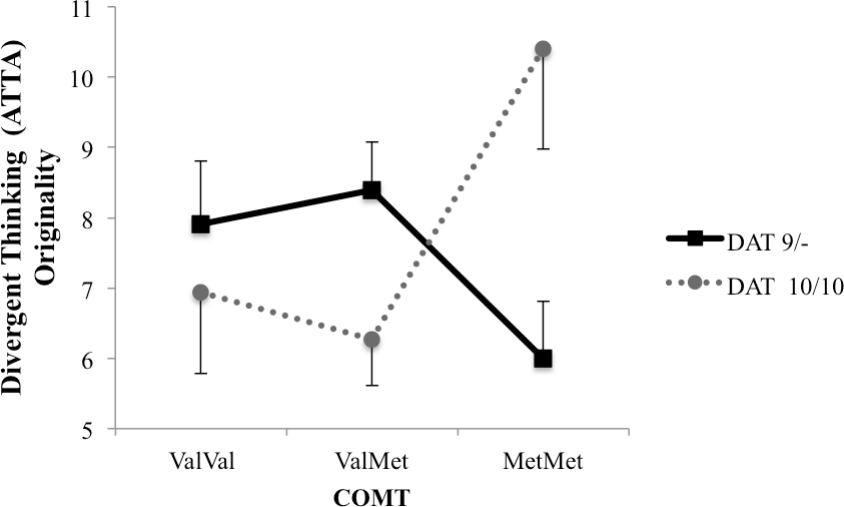
Estimated marginal means for ATTA originality scores, controlling for academic achievement.

Additional two separate 2 x 3 ANCOVAs were performed on participants’ ATTA fluency and ATTA originality scores, with academic achievement scores as a covariate. For fluency, there were no main effects, nor interactions, *p*s >. 36, suggesting that there is no association between the fluency factor of the ATTA and COMT, DAT, or COMT x DAT. For originality, there were no main effects, *p*s > .58, but there was a significant DAT x COMT interaction, *F*(2, 67) = 5.85, *p* = .005, partial Eta^2^ = .16. As can be seen from Fig 4, participants with the DAT 10/10 allele and COMT Met/Met allele, reflecting weak cognitive flexibility and strong top-down control, had the highest ATTA originality scores (*M* = 10.40, *SE* = 1.43).

### Results Involving Creative Achievement (CAQ) Performance and Dopaminergic Polymorphisms

**Fig 5:**
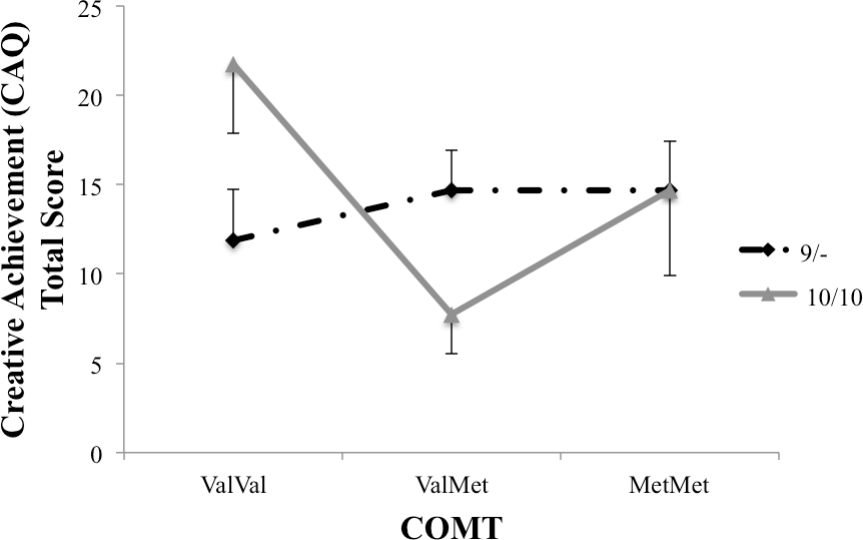
Estimated marginal means for creative achievement (CAQ) total scores, controlling for academic achievement.

A 2 (DAT: 9/-, 10/10) x 3 (COMT: ValVal, ValMet, MetMet) ANCOVA was calculated on participants’ creative achievement scores, with academic achievement scores as a covariate. There were no main effects for striatal (DAT) or frontal (COMT) dopamine, *p*s > .13, but there was a significant DAT x COMT interaction, *F*(2, 69) = 4.21, *p* = .02, partial Eta^2^ = .12. As can be seen from Fig 5, participants with DAT 10/10 and COMT ValVal, reflecting weak cognitive flexibility and weak top-down control, had the highest number of real-world creative achievements (*M* = 21.75, *SE =* 3.87). Post hoc comparisons using the Tukey’s HSD test indicated that the mean creative achievement score for the 10/10,ValVal group (*M* = 21.75, *SE* = 3.87) was reliably different from the means creative achievement score of the 10/10,Val/Met group (*M* = 7.74, *SE* = 2.20). A less stringent post hoc test using LSD Student’s *t*-test indicated that the mean creative achievement score for the 10/10,ValVal group (*M* = 21.75, *SE* = 3.87) was reliably different from the 9/-, ValMet group (*M* = 14.67, *SE* = 2.25); the mean creative achievement score for the 10/10,ValMet group (*M* = 7.74, *SE* = 2.20) was reliably different from the 9/-, ValMet group (*M* = 14.67, *SE* = 2.25), and the mean creative achievement score for the 10/10, ValMet group (*M* = 7.74, *SE* = 2.20) was reliably different from the 10/10, ValVal group (*M* = 21.75, *SE* = 3.87).

We also performed a 2 (DAT: 9/-, 10/10) x 3 (COMT: ValVal, ValMet, MetMet) ANOVA on participants’ creative achievement scores, *without* academic achievement scores as a covariate. There were no main effects for striatal (DAT) or frontal (COMT) dopamine, *p*s > .35, but there was a significant DAT x COMT interaction, *F*(2, 94) = 3.16, *p* < .05, partial Eta^2^ = .06. Total creative achievement means and standard deviations (not controlling for academic achievement) are presented in Table 3.

**Table 3:** Creative Achievement (CAQ) total score means and standard deviations for the COMT and DAT groups.

COMT	DAT	Mean	SD	N
**ValVal**	9/-	13.13	11.75	16
	10/10	17.64	11.20	11
	Total	14.96	11.63	27
**ValMet**	9/-	18.04	14.73	28
	10/10	9.08	7.08	25
	Total	13.81	12.50	53
**MetMet**	9/-	14.00	8.72	14
	10/10	11.33	10.56	6
	Total	13.20	9.12	20
**Total**	9/-	15.71	12.72	58
	10/10	11.64	9.34	42
	Total	14.00	11.56	100

As the CAQ data are generally positively skewed, we log-transformed total CAQ scores. The Kolmogorov-Smirnov test for normality indicated that the log-transformed CAQ distribution did not deviate significantly from a normal distribution (*D* = .08, *p* = .13). We again performed a 2 (DAT: 9/-, 10/10) x 3 (COMT: ValVal, ValMet, MetMet) ANCOVA on the log-transformed creative achievement scores, with academic achievement scores as a covariate. There was a marginally significant main effect for COMT, *F*(2,69) = 2.96, *p* = .06, partial Eta^2^ = .09, no main effects for DAT, and a significant DAT x COMT interaction, *F*(2, 69) = 5.20, *p* = .008, partial Eta^2^ = .14.

We performed similar analyses excluding participants with the mood disorders (*N* = 4), with the DAT x COMT interaction now at *F*(2, 65) = 5.99, *p* = .004, partial Eta^2^ = .17, a main effect for COMT, *F*(2,65) = 3.77, *p* = .03, partial Eta^2^ = .11, and no main effect for DAT, *p* > .93. Post hoc comparisons of the COMT main effect using Tukey’s HSD test indicated that the ValVal group (*M* = 16.95, SE = 2.51) was reliably different from the ValMet group (*M* = 10.43, SE = 1.57), indicating that the highest levels of creative achievements were in people with the weakest frontal top-down control.

**Fig 6:**
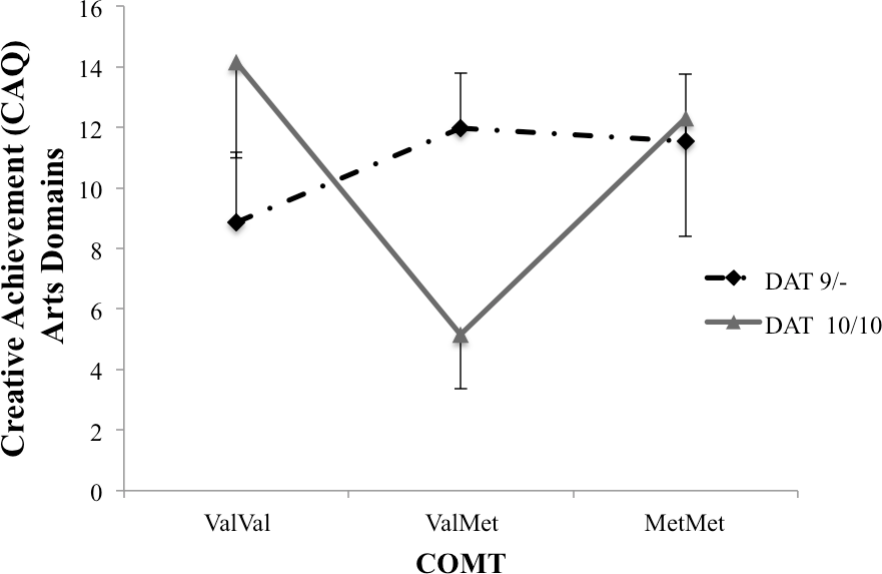
Estimated marginal means for creative achievements (CAQ) in the arts domains, controlling for academic achievement.

Examining creative achievements within arts and science domains separately, we found a DAT x COMT interaction predicting creative achievements in the arts, *F*(2, 94) = 5.56, *p* = .006, partial Eta^2^ = .15, but not in the science domains *p* > .73 (see Fig 6 for details). There were no main effects, *p*s > .10.

In MANCOVA, with the ATTA and CAQ as DVs, and COMT and DAT as IVs, with SAT/ACT scores as a covariate, the COMT x DAT interaction remained significant for the two latent variables (ATTA and CAQ) as a group, Wilks’ Lambda = .003. Univariate analyses further indicated a significant COMT x DAT interaction for ATTA, *F* = 4.28, *p* = .02, and a significant COMT x DAT interaction for CAQ, *F* = 4.26, *p* = .02 (CAQ log *F* = 5.28, *p* = .008). There were no main effects, *p*s > .42.

## Discussion

The aim of our study was to investigate whether two creativity measures–divergent thinking and creative achievement, can be predicted from genetic predispositions related to frontal and striatal dopaminergic functioning. Before we try to characterize the particular genetic profiles and their implications for creativity, we note that the overall pattern of the outcomes allow us to draw two conclusions. First, it was only particular interactions between the two genes we considered that were related to creativity, while there was little evidence for main effects. This means that there is unlikely to be one creativity gene that can explain individual differences in creative performance. Rather, it seems to be the interaction between dopaminergic pathways that relates to such individual differences. Of course there are likely to be other genes and factors that contribute to creative thinking. The finding of interactions in the absence of main effects also means that previous failures to find main effects of particular genes or polymorphisms must be interpreted with caution, as it may well be that their impact only unfolds in interaction with other genes.

Second, the interactions we obtained were different in kind for the two creativity measures. This suggests that the two measures are clearly indexing different aspects of creativity. In the present study this also seems apparent from the lack of a correlation between ATTA and CAQ scores, but some previous studies have reported small, but reliable correlations [47]. We take our present findings to show that the connection is not only weak, but the two tests can in fact be functionally double-dissociated to some degree.

Results from the ATTA do not only show that DAT and COMT interact, but also that they do so in opposite ways, depending on the DAT polymorphism: while the 9/- group shows the best ATTA performance in combination with ValMet, the polymorphism associated with medium top-down control, the 10/10 group shows peak performance in combination with MetMet, the polymorphism associated with particularly strong top-down control. If we consider the previous observations that the 9-repeat allele is related to various indications of good cognitive flexibility, while the 10-repeat allele is related to low learning abilities and ADHD, this pattern makes sense. The ATTA requires individuals to find new solutions and original answers, requiring some top-down guidance. It also considers the role of flexibility, which fits rather well with our observation that the performance of individuals with a genetic setup that supports cognitive flexibility (the 9/-carriers) benefit most from frontal top-down control that is effective, but not overly strong. Individuals with a less flexibility-supportive genetic setup, however, presumably require strong frontal guidance to overcome or compensate for the lack of flexibility. These findings are in line with the dual pathway to creativity model, which proposes that generation of original and appropriate ideas can indeed be achieved through either cognitive flexibility or through cognitive persistence [64].

The DAT x COMT interaction (weak cognitive flexibility and strong top-down control) was specific to the originality, but not the fluency factor of divergent thinking. Our results replicate previously reported DAT x COMT interaction on verbal originality of divergent thinking [9], although it is impossible to determine whether the specific nature of the interaction is replicated, given that post-hoc tests were not reported. There are also clear differences between our results and those in prior literature. Murphy and colleagues [9], for example, found a COMT x DAT interaction on the fluency of the divergent thinking, while Zhang and colleagues [10] reported three- and four-way interactions between variants of COMT and DRD2 on fluency and flexibility, but not on the originality of divergent thinking. Thus further investigations with larger sample sizes across various ethnic populations are needed to obtain a clear view of the role of DA transmission in divergent thinking.

Results from the CAQ suggest a particular benefit for 10-repeat carriers associated with weak cognitive flexibility, in combination with the ValVal polymorphism associated with particularly weak frontal top-down control. Although the link between creative achievement and DA transmission has not been previously investigated (but it has been proposed [65]), this observation fits well with previous reports that excellent performance on the CAQ is associated with “leaky” attention [47], with electrophysiological indications of reduced sensory gating [46], and with low latent inhibition [52]. As pointed out above, leaky attention may help individuals to take into consideration nominally irrelevant information, and integrate it with relevant information to create new ideas and insights. Not all creative achievements are alike, however–creative achievements in the arts, for example, may require, and in fact allow for more leaky attention than creative achievements in the science domains. In support, we found that the DAT x COMT interaction was significant specifically for participants with creative achievements in the arts, but not in the science domains.

Leaky attention may also make people more distractible or sensitive to environmental stimuli [65, 66], and has been linked with various types of psychopathology, particularly schizophrenia [67]. The interaction that we found might suggest that low DA levels would be potentially dysfunctional if not countered by high DA elsewhere in the brain.

In summary, our study suggests that divergent thinking and creative achievement can be predicted from interactions between genetic polymorphisms related to frontal and striatal dopaminergic pathways. Previous work similarly reported interactions between dopaminergic genes predicting various aspects of divergent thinking [9, 10]. Here, we find that different patterns were obtained for ATTA and CAQ, suggesting that these two measures tap into different aspects of creativity. It appears that successful performance on the divergent thinking tests generally benefits from a combination of good cognitive flexibility and medium top-down control, or from weak cognitive flexibility and strong top-down control. People with high real-world creative achievements, however, appear to have weak cognitive flexibility and weak top-down control.

Given that most creativity-training programs have a one-size-fits-all design, and assume that everyone benefits from interventions more or less in the same way, and more or less to the same degree, our results are quite relevant. In fact our outcome may suggest that efficiency of creativity training will be modulated by inter-individual differences with a genetic basis. Accordingly, only creativity programs that are tailored to individual abilities, skills, and needs are likely to succeed.

Our sample was somewhat limited, yielding low number in some of the subgroups, thus the results should be interpreted cautiously, but they warrant further study. Also, our use of rather highly aggregated assessments of creative achievement makes it difficult or impossible to identify particular processes involved in generating creative thoughts—for this purpose more process-pure measures would be more adequate, which, however, have the obvious drawback of being more remote from the eventual behavior. Finally, creativity measures in this study serve as a proxy for creativity, and various other measures of creativity exists. Future work will need to investigate how creativity as assessed by other creativity measures is linked with dopamine.

Taken altogether, our findings support the idea that dopamine levels in multiple brain areas affect human creativity, with an interaction between frontal and striatal dopaminergic pathways. As we have pointed out, this suggests a more cautious interpretation of previous failures to find main effects of particular genes, especially single genes analyzed in isolation, and even more so if only one measure of creativity was employed.
